# Supplementary material for: Do mothers pick up a phone? A cross-sectional study on delivery of MCH voice messages in Lagos, Nigeria
Source: PLoS One. 2022 Nov 7;17(11):e0275855. doi: 10.1371/journal.pone.0275855 (PMC9639833; doi:10.1371/journal.pone.0275855)
Supplement: Attachment — Submitted filename: PONE-D-22-00657_R1.docx [file pone.0275855.s003.docx]

**Manuscript ID:** PONE-D-22-00657_R1

**Title:** Do mothers pick up a phone? A cross-sectional study on delivery of MCH voice messages in Lagos, Nigeria

This is an important study that evaluates the extent to which voice message service users pick up the phone calls of voice messages and complete listening up to or beyond the core part of the voice messages. Given the ubiquitousness of mobile phones and the relatively low-cost of this intervention, the results of this study could guide resource-limited countries to an easily implementable intervention to improve maternal and child health utilization.

**Title**

Earlier reviewer’s comment and authors response noted

**Abstract**

Earlier reviewer’s comment and authors response noted. Revised abstract is well written and appropriately describes study’s key findings.

**Introduction**

The introduction carefully lists studies demonstrating the effectiveness of SMS and voice message for various maternal and child health services. For mHealth interventions to encourage action by recipients, the reminders, whether SMS, voice messages, or phone calls, first need to be acceptable. Authors could consider including a sentence or two on the acceptability of mobile phone messaging for maternal and child health. A cursory literature search showed a recent systematic review demonstrating overall acceptability of mobile phone reminders in Nigeria.

**Methods**

Earlier reviewers’ comments and authors response noted.

Authors could consider including (or explaining omission of) mothers’ parity in the models as high parity mothers with longer engagement with the maternal and child healthcare service would have different response compared to nulliparous mothers.

**Results**

Earlier reviewers’ comments and authors response noted.

**Discussion & conclusion**

Earlier reviewers’ comments and authors response noted. Authors revised discussion and English language edit was satisfactory.
