# Supplementary material for: Do mothers pick up a phone? A cross-sectional study on delivery of MCH voice messages in Lagos, Nigeria
Source: PLoS One. 2022 Nov 7;17(11):e0275855. doi: 10.1371/journal.pone.0275855 (PMC9639833; doi:10.1371/journal.pone.0275855)
Supplement: Attachment — Submitted filename: Response to Reviewers.docx [file pone.0275855.s004.docx]

**Response to Reviewers**

**Journal Requirements:**

Please review your reference list to ensure that it is complete and correct. If you have cited papers that have been retracted, please include the rationale for doing so in the manuscript text, or remove these references and replace them with relevant current references. Any changes to the reference list should be mentioned in the rebuttal letter that accompanies your revised manuscript. If you need to cite a retracted article, indicate the article’s retracted status in the References list and also include a citation and full reference for the retraction notice.

**Response:**

We appreciate the editor’s comment. The reference list has been corrected as follows.

| **#** | **Before** | **After** |
| --- | --- | --- |
| 1 | World Health Organization. mHealth: new horizons for health through mobile technologies: second global survey on eHealth [Internet]. World Health Organization; 2011 [cited 2021 Feb 16]. Available from: https://apps.who.int/iris/handle/10665/44607 | World Health Organization. mHealth: new horizons for health through mobile technologies: second global survey on eHealth. World Health Organization; 2011. Available: https://apps.who.int/iris/handle/10665/44607 |
| 2 | Organization WH, Others. Guidelines for treatment of drug-susceptible tuberculosis and patient care. 2017. Available: https://apps.who.int/iris/bitstream/handle/10665/255052/9789241550000-eng.pdf | World Health Organization. Guidelines for Treatment of Drug-Susceptible Tuberculosis and Patient Care, 2017 Update. World Health Organization; 2017. Accessed September 8, 2022. https://apps.who.int/iris/handle/10665/255052 |
| 4 | Rodrigues R, Shet A, Antony J, Sidney K, Arumugam K, Krishnamurthy S, et al. Supporting adherence to antiretroviral therapy with mobile phone reminders: results from a cohort in South India. PLoS One [Internet]. 2012 Aug 27;7(8):e40723. Available from: http://dx.doi.org/10.1371/journal.pone.0040723 | Rodrigues R, Shet A, Antony J, Sidney K, Arumugam K, Krishnamurthy S, et al. Supporting adherence to antiretroviral therapy with mobile phone reminders: results from a cohort in South India. PLoS One. 2012;7: e40723. doi:10.1371/journal.pone.0040723 |
| 5 | Rodrigues R, Poongulali S, Balaji K, Atkins S, Ashorn P, De Costa A. “The phone reminder is important, but will others get to know about my illness?” Patient perceptions of an mHealth antiretroviral treatment support intervention in the HIVIND trial in South India. BMJ Open [Internet]. 2015 Nov 2;5(11):e007574. Available from: http://dx.doi.org/10.1136/bmjopen-2015-007574 | Rodrigues R, Poongulali S, Balaji K, Atkins S, Ashorn P, De Costa A. “The phone reminder is important, but will others get to know about my illness?” Patient perceptions of an mHealth antiretroviral treatment support intervention in the HIVIND trial in South India. BMJ Open. 2015;5: e007574. doi:10.1136/bmjopen-2015-007574 |
| 6 | Piette JD, Mendoza-Avelares MO, Ganser M, Mohamed M, Marinec N, Krishnan S. A preliminary study of a cloud-computing model for chronic illness self-care support in an underdeveloped country. Am J Prev Med [Internet]. 2011 Jun;40(6):629–32. Available from: http://dx.doi.org/10.1016/j.amepre.2011.02.014 | Piette JD, Mendoza-Avelares MO, Ganser M, Mohamed M, Marinec N, Krishnan S. A preliminary study of a cloud-computing model for chronic illness self-care support in an underdeveloped country. Am J Prev Med. 2011;40: 629–632. doi:10.1016/j.amepre.2011.02.014 |
| 7 | Sambasivan N, Weber J, Cutrell E. Designing a phone broadcasting system for urban sex workers in India. In: Proceedings of the SIGCHI Conference on Human Factors in Computing Systems [Internet]. New York, NY, USA: Association for Computing Machinery; 2011 [cited 2021 Feb 12]. p. 267–76. (CHI ’11). Available from: https://doi.org/10.1145/1978942.1978980 | Sambasivan N, Weber J, Cutrell E. Designing a phone broadcasting system for urban sex workers in India. Proceedings of the SIGCHI Conference on Human Factors in Computing Systems. New York, NY, USA: Association for Computing Machinery; 2011. pp. 267–276. doi:10.1145/1978942.1978980 |
| 8 | Yamin F, Kaewkungwal J, Singhasivanon P, Lawpoolsri S. Women’s Perceptions of Using Mobile Phones for Maternal and Child Health Support in Afghanistan: Cross-Sectional Survey. JMIR Mhealth Uhealth [Internet]. 2018 Apr 10;6(4):e76. Available from: http://dx.doi.org/10.2196/mhealth.9504 | Yamin F, Kaewkungwal J, Singhasivanon P, Lawpoolsri S. Women’s Perceptions of Using Mobile Phones for Maternal and Child Health Support in Afghanistan: Cross-Sectional Survey. JMIR Mhealth Uhealth. 2018;6: e76. doi:10.2196/mhealth.9504 |
| 9 | Lebrun V, Dulli L, Alami SO, Sidiqi A, Sultani AS, Rastagar SH, et al. Feasibility and Acceptability of an Adapted Mobile Phone Message Program and Changes in Maternal and Newborn Health Knowledge in Four Provinces of Afghanistan: Single-Group Pre-Post Assessment Study. JMIR Mhealth Uhealth [Internet]. 2020 Jul 20;8(7):e17535. Available from: http://dx.doi.org/10.2196/17535 | Lebrun V, Dulli L, Alami SO, Sidiqi A, Sultani AS, Rastagar SH, et al. Feasibility and Acceptability of an Adapted Mobile Phone Message Program and Changes in Maternal and Newborn Health Knowledge in Four Provinces of Afghanistan: Single-Group Pre-Post Assessment Study. JMIR Mhealth Uhealth. 2020;8: e17535. doi:10.2196/17535 |
| 10 | Pai N, Supe P, Kore S, Nandanwar YS, Hegde A, Cutrell E, et al. Using automated voice calls to improve adherence to iron supplements during pregnancy: a pilot study. In: Proceedings of the Sixth International Conference on Information and Communication Technologies and Development: Full Papers - Volume 1 [Internet]. New York, NY, USA: Association for Computing Machinery; 2013 [cited 2021 Feb 15]. p. 153–63. (ICTD ’13). Available from: https://doi.org/10.1145/2516604.2516608 | Pai N, Supe P, Kore S, Nandanwar YS, Hegde A, Cutrell E, et al. Using automated voice calls to improve adherence to iron supplements during pregnancy: a pilot study. Proceedings of the Sixth International Conference on Information and Communication Technologies and Development: Full Papers - Volume 1. New York, NY, USA: Association for Computing Machinery; 2013. pp. 153–163. doi:10.1145/2516604.2516608 |
| 11 | Downs SM, Sackey J, Kalaj J, Smith S, Fanzo J. An mHealth voice messaging intervention to improve infant and young child feeding practices in Senegal. Matern Child Nutr [Internet]. 2019 Oct;15(4):e12825. Available from: http://dx.doi.org/10.1111/mcn.12825 | Downs SM, Sackey J, Kalaj J, Smith S, Fanzo J. An mHealth voice messaging intervention to improve infant and young child feeding practices in Senegal. Matern Child Nutr. 2019;15: e12825. doi:10.1111/mcn.12825 |
| 12 | Kruse C, Betancourt J, Ortiz S, Valdes Luna SM, Bamrah IK, Segovia N. Barriers to the Use of Mobile Health in Improving Health Outcomes in Developing Countries: Systematic Review. J Med Internet Res [Internet]. 2019 Oct 9;21(10):e13263. Available from: http://dx.doi.org/10.2196/13263 | Kruse C, Betancourt J, Ortiz S, Valdes Luna SM, Bamrah IK, Segovia N. Barriers to the Use of Mobile Health in Improving Health Outcomes in Developing Countries: Systematic Review. J Med Internet Res. 2019;21: e13263. doi:10.2196/13263 |
| 13 | Okuboyejo S, Omoregbe NA, Mbarika V. A Framework for the Design of a Mobile-Based Alert System for Outpatient Adherence in Nigeria. African Journal of Computing & ICT. 2012;5: 151–158. Available: http://eprints.covenantuniversity.edu.ng/765/ | Okuboyejo S, Omoregbe NA, Mbarika V. A Framework for the Design of a Mobile-Based Alert System for Outpatient Adherence in Nigeria. African Journal of Computing & ICT. 2012;5: 151–158. Available: https://afrjcict.net/wp-content/uploads/2017/08/vol-5-no-5-september-20121.pdf |
| 14 | Olajubu AO, Fajemilehin BR, Olajubu TO, Afolabi BS. Effectiveness of a mobile health intervention on uptake of recommended postnatal care services in Nigeria. PLoS One [Internet]. 2020 Sep 14;15(9):e0238911. Available from: http://dx.doi.org/10.1371/journal.pone.0238911 | Olajubu AO, Fajemilehin BR, Olajubu TO, Afolabi BS. Effectiveness of a mobile health intervention on uptake of recommended postnatal care services in Nigeria. PLoS One. 2020;15: e0238911. doi:10.1371/journal.pone.0238911 |
| 15 | Kawakatsu Y, Oyeniyi Adesina A, Kadoi N, Aiga H. Cost-effectiveness of SMS appointment reminders in increasing vaccination uptake in Lagos, Nigeria: A multi-centered randomized controlled trial. Vaccine [Internet]. 2020 Aug 9; Available from: http://dx.doi.org/10.1016/j.vaccine.2020.07.075 | Kawakatsu Y, Oyeniyi Adesina A, Kadoi N, Aiga H. Cost-effectiveness of SMS appointment reminders in increasing vaccination uptake in Lagos, Nigeria: A multi-centered randomized controlled trial. Vaccine. 2020. doi:10.1016/j.vaccine.2020.07.075 |
| 16 | Wynn R, Oyeyemi F. Giving cell phones to pregnant women and improving services may increase primary health facility utilization: a. 2014. Available: https://www.academia.edu/download/44533831/1742-4755-11-8.pdf | Oyeyemi SO, Wynn R. Giving cell phones to pregnant women and improving services may increase primary health facility utilization: a case-control study of a Nigerian project. Reprod Health. 2014;11: 8. doi:10.1186/1742-4755-11-8 |
| 17 | Tripathi V, Arnoff E, Bellows B, Sripad P. Use of interactive voice response technology to address barriers to fistula care in Nigeria and Uganda. Mhealth [Internet]. 2020 Apr 5;6:12. Available from: http://dx.doi.org/10.21037/mhealth.2019.12.04 | Tripathi V, Arnoff E, Bellows B, Sripad P. Use of interactive voice response technology to address barriers to fistula care in Nigeria and Uganda. Mhealth. 2020;6: 12. doi:10.21037/mhealth.2019.12.04 |
| 18 | Babalola S, Loehr C, Oyenubi O, Akiode A, Mobley A. Efficacy of a Digital Health Tool on Contraceptive Ideation and Use in Nigeria: Results of a Cluster-Randomized Control Trial. Glob Health Sci Pract [Internet]. 2019 Jun;7(2):273–88. Available from: http://dx.doi.org/10.9745/GHSP-D-19-00066 | Babalola S, Loehr C, Oyenubi O, Akiode A, Mobley A. Efficacy of a Digital Health Tool on Contraceptive Ideation and Use in Nigeria: Results of a Cluster-Randomized Control Trial. Glob Health Sci Pract. 2019;7: 273–288. doi:10.9745/GHSP-D-19-00066 |
| 19 | Chowdhury ME, Shiblee SI, Jones HE. Does mHealth voice messaging work for improving knowledge and practice of maternal and newborn healthcare? BMC Med Inform Decis Mak [Internet]. 2019 Sep 5;19(1):179. Available from: http://dx.doi.org/10.1186/s12911-019-0903-z | Chowdhury ME, Shiblee SI, Jones HE. Does mHealth voice messaging work for improving knowledge and practice of maternal and newborn healthcare? BMC Med Inform Decis Mak. 2019;19: 179. doi:10.1186/s12911-019-0903-z |
| 20 | Murthy N, Chandrasekharan S, Prakash MP, Ganju A, Peter J, Kaonga N, et al. Effects of an mHealth voice message service (mMitra) on maternal health knowledge and practices of low-income women in India: findings from a pseudo-randomized controlled trial. BMC Public Health [Internet]. 2020 Jun 1;20(1):820. Available from: http://dx.doi.org/10.1186/s12889-020-08965-2 | Murthy N, Chandrasekharan S, Prakash MP, Ganju A, Peter J, Kaonga N, et al. Effects of an mHealth voice message service (mMitra) on maternal health knowledge and practices of low-income women in India: findings from a pseudo-randomized controlled trial. BMC Public Health. 2020;20: 820. doi:10.1186/s12889-020-08965-2 |
| 21 | Murthy N, Chandrasekharan S, Prakash MP, Kaonga NN, Peter J, Ganju A, et al. The Impact of an mHealth Voice Message Service (mMitra) on Infant Care Knowledge, and Practices Among Low-Income Women in India: Findings from a Pseudo-Randomized Controlled Trial. Matern Child Health J [Internet]. 2019 Dec;23(12):1658–69. Available from: http://dx.doi.org/10.1007/s10995-019-02805-5 | Murthy N, Chandrasekharan S, Prakash MP, Kaonga NN, Peter J, Ganju A, et al. The Impact of an mHealth Voice Message Service (mMitra) on Infant Care Knowledge, and Practices Among Low-Income Women in India: Findings from a Pseudo-Randomized Controlled Trial. Matern Child Health J. 2019;23: 1658–1669. doi:10.1007/s10995-019-02805-5 |
| 22 | DeSouza SI, Rashmi MR, Vasanthi AP, Joseph SM, Rodrigues R. Mobile phones: the next step towards healthcare delivery in rural India? PLoS One [Internet]. 2014 Aug 18;9(8):e104895. Available from: http://dx.doi.org/10.1371/journal.pone.0104895 | DeSouza SI, Rashmi MR, Vasanthi AP, Joseph SM, Rodrigues R. Mobile phones: the next step towards healthcare delivery in rural India? PLoS One. 2014;9: e104895. doi:10.1371/journal.pone.0104895 |
| 23 | Huang S, Li M. Piloting a mHealth intervention to improve newborn care awareness among rural Cambodian mothers: a feasibility study. BMC Pregnancy Childbirth [Internet]. 2017 Oct 16;17(1):356. Available from: http://dx.doi.org/10.1186/s12884-017-1541-z | Huang S, Li M. Piloting a mHealth intervention to improve newborn care awareness among rural Cambodian mothers: a feasibility study. BMC Pregnancy Childbirth. 2017;17: 356. doi:10.1186/s12884-017-1541-z |
| 24 | Amankwaa I, Boateng D, Quansah DY, Akuoko CP, Evans C. Effectiveness of short message services and voice call interventions for antiretroviral therapy adherence and other outcomes: A systematic review and meta-analysis. PLoS One [Internet]. 2018 Sep 21;13(9):e0204091. Available from: http://dx.doi.org/10.1371/journal.pone.0204091 | Amankwaa I, Boateng D, Quansah DY, Akuoko CP, Evans C. Effectiveness of short message services and voice call interventions for antiretroviral therapy adherence and other outcomes: A systematic review and meta-analysis. PLoS One. 2018;13: e0204091. doi:10.1371/journal.pone.0204091 |
| 25 | Crawford J, Larsen-Cooper E, Jezman Z, Cunningham SC, Bancroft E. SMS versus voice messaging to deliver MNCH communication in rural Malawi: assessment of delivery success and user experience. Glob Health Sci Pract [Internet]. 2014 Feb;2(1):35–46. Available from: http://dx.doi.org/10.9745/GHSP-D-13-00155 | Crawford J, Larsen-Cooper E, Jezman Z, Cunningham SC, Bancroft E. SMS versus voice messaging to deliver MNCH communication in rural Malawi: assessment of delivery success and user experience. Glob Health Sci Pract. 2014;2: 35–46. doi:10.9745/GHSP-D-13-00155 |
| 26 | LeFevre AE, Mohan D, Hutchful D, Jennings L, Mehl G, Labrique A, et al. Mobile Technology for Community Health in Ghana: what happens when technical functionality threatens the effectiveness of digital health programs? BMC Med Inform Decis Mak [Internet]. 2017 Mar 14;17(1):27. Available from: http://dx.doi.org/10.1186/s12911-017-0421-9 | LeFevre AE, Mohan D, Hutchful D, Jennings L, Mehl G, Labrique A, et al. Mobile Technology for Community Health in Ghana: what happens when technical functionality threatens the effectiveness of digital health programs? BMC Med Inform Decis Mak. 2017;17: 27. doi:10.1186/s12911-017-0421-9 |
| 27 | Anastasi E, Ekanem E, Hill O, Adebayo Oluwakemi A, Abayomi O, Bernasconi A. Unmasking inequalities: Sub-national maternal and child mortality data from two urban slums in Lagos, Nigeria tells the story. PLoS One [Internet]. 2017 May 10;12(5):e0177190. Available from: http://dx.doi.org/10.1371/journal.pone.0177190 | Anastasi E, Ekanem E, Hill O, Adebayo Oluwakemi A, Abayomi O, Bernasconi A. Unmasking inequalities: Sub-national maternal and child mortality data from two urban slums in Lagos, Nigeria tells the story. PLoS One. 2017;12: e0177190. doi:10.1371/journal.pone.0177190 |
| 28 | National Population Commission (NPC) [Nigeria] and ICF. Nigeria Demographic and Health Survey 2018. Abuja, Nigeria, and Rockville, Maryland, USA: NPC and ICF.; 2019. Available: https://dhsprogram.com/pubs/pdf/FR359/FR359.pdf | National Population Commission (NPC), ICF. Nigeria Demographic and Health Survey 2018. Abuja, Nigeria, and Rockville, Maryland, USA: NPC and ICF.; 2019. Available: http://dhsprogram.com/pubs/pdf/FR359/FR359.pdf. |
| 29 | United Nations Publications. World Urbanization Prospects: The 2018 Revision. UN; 2019. Available: https://play.google.com/store/books/details?id=Kp9AygEACAAJ | United Nations, Department of Economic and Social Affairs, Population Division. World Urbanization Prospects: The 2018 Revision (ST/ESA/SER.A/420). New York: United Nations; 2019. Available: https://population.un.org/wup/publications/Files/WUP2018-Report.pdf |
| 30 | Lagos bureau of Statistics, Ministry of Economic Planning and Budget. Abstract of Local Government Statistics [Internet]. Lagos State Government; 2016. Available from: https://mepb.lagosstate.gov.ng/wp-content/uploads/sites/29/2017/01/ABSTRACT-OF-LOCAL-GOVERNMENT-STATISTICS-2016.pdf | Lagos bureau of Statistics, Ministry of Economic Planning and Budget. Abstract of Local Government Statistics. Lagos State Government; 2016. Available: https://mepb.lagosstate.gov.ng/wp-content/uploads/sites/29/2017/01/ABSTRACT-OF-LOCAL-GOVERNMENT-STATISTICS-2016.pdf |
| 31 | Danladi SS. Language policy: Nigeria and the role of English language in the 21st century. European Scientific Journal. 2013. Available: https://core.ac.uk/download/pdf/328023643.pdf | Danladi SS. Language policy: Nigeria and the role of English language in the 21st century. European Scientific Journal. 2013;9. Available: https://eujournal.org/index.php/esj/article/view/1153 |
| 32 | Filmer D, Pritchett LH. Estimating wealth effects without expenditure data--or tears: an application to educational enrollments in states of India. Demography [Internet]. 2001 Feb;38(1):115–32. Available from: https://read.dukeupress.edu/demography/article/38/1/115/170414/Estimating-wealth-effects-without-expenditure-data | Filmer D, Pritchett LH. Estimating wealth effects without expenditure data—or tears: an application to educational enrollments in states of India. Demography. 2001;38: 115–132. doi:10.1353/dem.2001.0003 |
| 33 | Kimani-Murage EW, Wekesah F, Wanjohi M, Kyobutungi C, Ezeh AC, Musoke RN, et al. Factors affecting actualisation of the WHO breastfeeding recommendations in urban poor settings in Kenya. Matern Child Nutr [Internet]. 2015 Jul;11(3):314–32. Available from: https://onlinelibrary.wiley.com/doi/10.1111/mcn.12161 | Kimani-Murage EW, Wekesah F, Wanjohi M, Kyobutungi C, Ezeh AC, Musoke RN, et al. Factors affecting actualisation of the WHO breastfeeding recommendations in urban poor settings in Kenya. Matern Child Nutr. 2015;11: 314–332. doi:10.1111/mcn.12161 |
| 34 | Piette JD, Valverde H, Marinec N, Jantz R, Kamis K, de la Vega CL, et al. Establishing an independent mobile health program for chronic disease self-management support in bolivia. Front Public Health [Internet]. 2014 Aug 13;2:95. Available from: http://dx.doi.org/10.3389/fpubh.2014.00095 | Piette JD, Valverde H, Marinec N, Jantz R, Kamis K, de la Vega CL, et al. Establishing an independent mobile health program for chronic disease self-management support in Bolivia. Front Public Health. 2014;2: 95. doi:10.3389/fpubh.2014.00095 |
